# Decolonising women’s health innovation

**DOI:** 10.1136/bmj-2025-085683

**Published:** 2025-10-10

**Authors:** Tiffany Nassiri-Ansari, Alana Gall, Samuel Oji Oti, Sawsan Abdulrahim, Emma L M Rhule

**Affiliations:** 1 United Nations University International Institute for Global Health, Kuala Lumpur, Malaysia; 2 Hibiscus Horizons, Kuala Lumpur, Malaysia; 3 National Centre for Naturopathic Medicine, Southern Cross University, Lismore, Australia; 4 Traditional, Complementary and Integrative Healthcare Coalition, Brussels, Belgium; 5 Global Health Decolonisation Movement in Africa, Nairobi, Kenya; 6 Faculty of Health Sciences, American University of Beirut, Beirut, Lebanon

## Abstract

**Tiffany Nassiri-Ansari and colleagues** set out how a decolonial feminist approach to innovation could produce greater gender equality and health equity

The field of women’s health innovation is growing rapidly, yet it has focused mainly on technological solutions. Despite the global scope and ambition of advocates and innovators, feminist and decolonial perspectives remain largely absent. These perspectives are vital to discover whose voices and priorities shape innovation, and to improve how the field allocates funding, directs investments, and ensures inclusive representation and leadership.

For too long, communities have had “innovative” solutions imposed on them under the banners of novelty and progress, without regard for contextual fit^1^ or alignment with local priorities.^2^ In addition to being a potential waste of often scarce resources, such interventions often reinforce power imbalances, sustaining top-down decision making and restricting opportunities for affected communities to shape the solutions that affect them. In many cases they have perpetuated, rather than dismantled, the inequities they claim to tackle.

Centring decolonial feminist values in gender and health innovation is not a new idea. Where activists, community leaders, researchers, and funders have rejected exclusionary, top-down processes in favour of collective, inclusive ones, tangible improvements in health and wellbeing have followed.^3^ Building on that tradition, this article, part of the BMJ Collection on Women’s Health Innovation (bmj.com/collections/womens-health-innovation), calls for a deliberate reimagining of how women’s health innovation is defined, developed, and delivered.

To understand why such a shift is necessary, it is essential to consider how the enduring structures and logics of coloniality shape health systems, research, and innovation and, in turn, the health and wellbeing of women and gender diverse people worldwide.

## Coloniality as a social and structural determinant of health

Coloniality refers to the enduring systems of power, knowledge, and being that emerged from colonisation and which continue to shape the modern world. These structures extend far beyond formal colonial rule, operating through economic arrangements, political institutions, cultural norms, and knowledge systems that privilege certain worldviews while marginalising others.

In the context of health, coloniality functions as a deep rooted social and structural determinant.^4^ ^5^ It shapes which health issues receive attention and resources, whose knowledge is considered legitimate, and who holds decision making power. For women and gender diverse people, especially those in formerly colonised regions, this often means facing intersecting forms of discrimination based on factors including, but not limited to, gender, race, class, and geography.

The effects are both material and epistemic. Materially, coloniality entrenches economic inequities, limiting access to resources, infrastructure, and services. Epistemically, it devalues indigenous, local, and experiential knowledge, favouring biomedical and technological approaches developed in high income settings. These dynamics are often reproduced in global health innovations that fail to critically examine their underlying assumptions and power structures, even in initiatives that claim to be inclusive or equitable.

Recognising coloniality as a determinant of health makes visible the historical and systemic forces that shape inequities. It also opens the door to transformative approaches that centre the voices, priorities, and leadership of those most affected, an imperative at the heart of a decolonial feminist vision for women’s health innovation.

## Limits of technological innovation

From life saving vaccines and novel pharmaceuticals to medical imaging and artificial organs, technological innovation has played an important part in driving improved health outcomes and tackling persistent inequities.^6^ However, dominant understandings of innovation tend to privilege technological breakthroughs and market driven solutions, often at the expense of social, cultural, and indigenous approaches. These technology and business centric models often fail to tackle the root causes of many health inequities.^7^ They frequently overlook the complex social determinants shaping gendered and racialised disparities, and risk reinforcing existing inequalities, producing innovations that are ineffective at best and actively harmful at worst.

In women’s health, innovation has traditionally focused on developing technologies and products to diagnose and treat conditions—for example, the Gates Foundation’s recent $2.5bn (£1.9bn; €2.1bn) investment into 40 priority innovations, ranging from new contraceptive methods to maternal health diagnostics.^8^ While such investments are important, innovation must also include approaches that tackle the structural drivers of inequity, including coloniality and patriarchy. Without this broader scope, innovation risks entrenching the very power imbalances it should be dismantling.

As others have argued, innovation in women’s health must extend beyond technological solutions.^9^ ^10^ Social innovation, “a novel solution to a social problem that is more effective, sustainable, or just than existing solutions and for which the value created accrues primarily to society as a whole rather than private individuals,”^11^ has been put forward as one solution to these problems. Innovations need not be new inventions or wholly original to be considered novel; rather, they are often “new to the user, context, or application”^7^ ^11^ and must create value, such as improved health outcomes, better access to services, or reduced costs. However, the practice of social innovation is not uniform. Divergent models have emerged, including technocratic, democratic, and institutional or structural approaches. Technocratic social innovation has been criticised as promoting the dominant neoliberal agenda and reinforcing top-down power. By contrast, democratic or inclusive social innovation models seek “to meet human needs by increasing participation levels and empowerment, enabling greater access to resources, and increasing social and political capacities.”^7^

However, social innovation that fails to explicitly confront intersecting power asymmetries, including coloniality and patriarchy, risks perpetuating the very inequities it aims to resolve. Incremental measures such as expanding participation and inclusion must not replace or displace deeper interrogations of the intersecting power asymmetries that underpin global health inequities.

To counter coloniality in health innovation, three fundamental questions must guide our approach:

- Who is recognised as an innovator, and whose voices are excluded? How do we raise the contributions of those historically marginalised, ensuring equitable inclusion and participation in a manner that is beyond consultation but is truly co-created and co-owned?
- What forms of innovation are privileged, and what alternatives are overlooked? How do we ensure that we are adopting an expansive definition of innovation that incorporates different ways of knowing and being, and recognises the value of social innovation and community grounded knowledge?
- Why do we innovate, and for whose benefit? How can we prioritise returns on investment that recognise health as both a right and a public good, and that benefit communities over shareholders, ensuring progress fosters collective wellbeing and sustainable change?

We argue that it is important to clearly recognise coloniality as both a racialised and gendered force, one that has shaped the global systems of power and identity, including how innovation is defined and practised. Ignoring this risks creating solutions that are restrictively narrow and unable to deal with the complexity of the challenges we face. A decolonial feminist lens that sees coloniality and patriarchy as deeply connected root causes helps us understand how even well meaning efforts to promote gender equality and health equity can sometimes reinforce the very asymmetries they aim to redress.

In this paper, we outline an approach to health innovation that reckons with coloniality as a determinant of health. We apply a decolonial feminist lens and share examples that illustrate how this lens can reshape participation, process, and prioritisation in women’s health innovation to advance more equitable health outcomes.

## Decolonial feminist lens for innovation

Patriarchal power structures today are deeply shaped by the legacies of the European colonial project, whose worldviews continue to influence societies long after formal colonisation ended. These enduring systems embed norms and associated power dynamics around gender, sexuality, race, and other identities, often reinforcing discrimination and harm. Tackling patriarchy without tackling coloniality is therefore futile; the two are inextricably intertwined.

Decolonial feminism examines how coloniality and patriarchy intersect with different systems of oppression, including racism, capitalism, ableism, and classism.^12^ ^13^ ^14^ ^15^ ^16^ Conceptualised as a tool for action, a decolonial feminism approach rejects siloed advocacy, instead calling for collective action across struggles that share structural roots.^17^ Although the focus is largely on the intersection of racism and sexism, decolonial feminism helps interrogate and confront power asymmetries across three key aspects of the innovation cycle—participation, process, and prioritisation—and in doing so, offers principles for making innovation products more inclusive and equitable for all. Adopting this approach can benefit the emerging and growing field and priorities of women’s health innovation.

### Participation

Innovation requires a deliberate shift from merely consulting with communities to actively centring community voices as experts. These processes must reject epistemic violence, the devaluation, distortion, and destruction of diverse ways of knowing, and be respectful of different expressions of knowing and being. They also need to expand the definition of “innovators” to include those with lived experience and to harness community knowledge to develop solutions, rather than gatekeeping the title for those with formal credentials, institutional affiliations, and theoretical knowledge.

The Amenah intervention (box 1), which has sought to tackle early marriage among Syrian refugee girls in Lebanon since 2017, piloted an innovative community method that trained and positioned community members as knowledge holders.^20^ Through centring community members as both researchers (trained facilitators) and participants (young girls), the project developed an intervention based on the experiences and needs of the girls themselves. Grounded in a destigmatising approach, it focused on harm reduction through official registration and recognition of early marriages, providing legal protection and recourse against the most severe health impacts of early marriage, including adolescent pregnancy and intimate partner violence.

Box 1 **Amenah intervention to tackle drivers of early marriage**

Early marriage exists at the intersection of gender and health, demonstrating how socioeconomic realities driven by patriarchy can lead to critical health challenges for young women and girls. Globally, one in five young women were married before the age of 18, placing them at increased risk of physical and sexual intimate partner violence and more likely to experience complications of pregnancy and childbirth.^18^

Despite surveys showing that the compounding vulnerabilities experienced by displaced, migrant, and refugee young women and girls put them at a higher risk of early marriage, few interventions are designed for or implemented among these populations. For instance, a 2016 survey of Syrian refugees in three Lebanese towns showed that nearly 25% of girls were married by the age of 18, with most having already experienced at least one pregnancy,^19^ but there is limited political will in Lebanon to deal with the issue at the national level. This reluctance can be partly attributed to a perception of early marriage as a patriarchal cultural practice that travelled with refugees, as opposed to a circumstantial response to the socioeconomic realities of displacement.

**Innovation**

First piloted in 2017, phase 1 of the Amenah (Arabic for girls/women who are secure and protected) project trained women from the community to deliver education sessions to young girls on topics including puberty and menstruation, gender equity and rights, and assertive communication and self-confidence, with complementary sessions for mothers. Community workers also served as knowledge brokers between researchers and the community, with whom the research team had no direct engagement.^20^

Building on studies that showed better uptake of messaging when delivered by someone with whom young people can identify, Amenah phase 2 saw an iterated approach, introducing “peer educators” (aged 18-early 20s) to lead the participatory sessions with young girls. The phase 1 community workers, who had developed strong relationships in the community, continued to facilitate sessions with the girls’ mothers. Additionally, a community advisory board consisting of people living or working in the community of interest was assembled to provide insight into community priorities, help deal with acceptability concerns, and enhance buy-in to promote sustainability.^21^

Findings from the project uncovered drivers of early marriage, including harassment and sexual violence perpetuated against women and girls, financial challenges, and societal norms related to family honour, informing “a new conceptualisation of early marriage as a social outcome rather than a stigmatising deficiency or a problem.”^20^ This shift enabled the implementation of practical harm reduction measures such as legally registering existing marriages.

**Outcomes**

Although official registration of early marriages might appear counterintuitive in that it makes binding that which might have otherwise been an informal partnership, this is an example of how centring the voices of those with lived experience can lead to more contextually relevant interventions. Official registration of marriages allows girls to access legal protection and recourse, with the aim of mitigating some of the worst outcomes of early marriage. Interventions such as these can contribute to lower rates of intimate partner violence, fewer cases of spousal abandonment, and improved sexual and reproductive health outcomes.

### Process

Innovation processes are not divorced from the broader social, economic, and political structures within which they are embedded and must be investigated for manifestations of bias, prejudice, and discrimination. The knowledge systems from which innovation processes draw largely reflect the hegemony of western and biomedical epistemologies. When processes do draw on or integrate other forms of evidence and expertise, many do so in an appropriative manner that fails to acknowledge the value of other ways of knowing and being, such as indigenous traditional knowledge. Such acts of epistemic injustice must be challenged, ensuring not only that diverse knowledge systems are recognised as valuable from the outset but also that they are credited when used.

The 2024 World Intellectual Property Organization (WIPO) Treaty on Intellectual Property, Genetic Resources and Associated Traditional Knowledge (box 2), which acknowledges indigenous knowledge as intellectual property for the first time in international law,^22^ is both an example of how processes can be reformed to be more inclusive and equitable and a resource for those looking to ensure their innovation practices tackle centuries of epistemic injustice perpetuated through medical research and development processes. Implementation of the treaty, co-led by national intellectual property agencies and indigenous communities, is projected to create more inclusive and informed innovation processes and products that truly advance the cause of health equity for all.

Box 2 **WIPO Treaty on Intellectual Property, Genetic Resources, and Associated Traditional Knowledge**

Coloniality has long waged a campaign of epistemicide against indigenous knowledge, committing acts of epistemic violence during colonisation and perpetuating devaluation-as-destruction in modern processes of knowledge generation and innovation. In research and development, this has largely manifested in the unacknowledged use (if not outright theft) of indigenous traditional knowledge as the basis of new medicines and other treatments, also known as biopiracy. While these practices generate profitable products and industries, they worsen health inequities by denying indigenous communities the credit and benefits for their wisdom. Negotiations for a treaty to recognise indigenous knowledge as intellectual property formally began at the World Intellectual Property Organization in 2001, “initiated in 1999 with a proposal by Colombia, where discussions were notable for their inclusion of indigenous peoples as well as local communities.”^22^

**Innovation**

The 2024 WIPO Treaty on Intellectual Property, Genetic Resources, and Associated Traditional Knowledge, driven in large part by the WIPO indigenous caucus, establishes “in international law a new disclosure requirement for patent applicants whose inventions are based on genetic resources and/or associated traditional knowledge.”^22^ It has the potential to actively reshape the broader systems and structures that direct innovation processes, such that diverse ways of knowing not only feature in research and development processes but benefit the communities whose knowledge enabled crucial breakthroughs. Active and meaningful participation of the indigenous caucus at treaty negotiations was made possible in part through WIPO’s ad hoc fast track accreditation procedure, a voluntary fund to support participation of accredited representatives, and indigenous panels that centre “the experiences, concerns and aspirations of Indigenous Peoples and local communities.”^23^

**Outcomes**

Given the recent adoption of the treaty and with member states still in the process of integrating the articles into national and regional law, it is too early to assess the treaty’s impact. Some national governments are beginning to explore legislative and administrative pathways to implement the treaty’s provisions. In Australia, IP Australia has established an indigenous knowledge panel to inform improvements to the intellectual property system, ensuring it better recognises and protects Aboriginal and Torres Strait Islander knowledge across all intellectual property categories—not just patents. In addition, the Australian Commonwealth Government is undergoing the development of standalone indigenous cultural and intellectual property legislation, led by Patricia Adjei and guided by a First Nations Expert Working Group. These initiatives reflect a broader shift toward embedding indigenous governance and self-determination in intellectual property reform. Projected outcomes include more inclusive and informed innovation processes for new therapeutics and greater acknowledgment of, and remuneration for, indigenous knowledge and expertise.

### Prioritisation

Adopting a decolonial feminist approach to prioritisation in innovation requires a re-evaluation of what is considered valuable and impactful, particularly by funders and financiers of innovation whose resources make them key power holders. It necessitates shifting the focus from traditional metrics of success, often defined by narrow quantitative indicators such as number of patents filed or shareholder profit, to context specific metrics focused on collective wellbeing, equity, and sustainability.^24^ This shift would impact innovation products and processes alike, challenging methodological colonialism and its emphasis on western knowledge creation modes and norms that may not serve community priorities. Innovation processes that embed or align with a decolonial feminist approach to focus on community needs and transformative outcomes are well placed to strengthen community agency, support local capacities, and contribute to intergenerational resilience.

The Trust-Based Philanthropy Project (box 3) is an example of a network of trust based funders realigning their priorities to better serve community needs. By providing long term, flexible, and core funding and reforming accountability and reporting mechanisms to be multi-directional and ultimately in the service of communities, trust based decolonial feminist philanthropy represents a structural shift in funding that not only prioritises but also enables shifts in power dynamics.^25^

Box 3 **Trust-Based Philanthropy Project**

Funding is a crucial element of the innovation process, as funders often have the power to influence, if not dictate, who is involved in the process, what counts as an innovation, and whose needs and which outcomes are prioritised. Some funders reinforce colonial power asymmetries through measures of success that do not reflect community priorities, timelines that encourage quick fixes rather than tackling root causes, and narrow accountability mechanisms that fail to serve those most in need.^25^

**Innovation**

In 2020, The Trust-Based Philanthropy Project started as a peer network to “promote a more equitable and relational approach to philanthropy.”^26^ Drawing on four key dimensions— culture, structures, leadership, and practices—trust based philanthropy calls for the adoption of “a values-driven approach that reimagines the role of funders as collaborators working alongside non-profit organisations, reinforcing collective accountability towards the communities they serve and support.”^25^ This approach trusts community knowledge, avoids reinforcing hierarchies, and promotes multidirectional accountability between all stakeholders through six practices: give multi-year, unrestricted funding; do the homework; simplify and streamline paperwork; be transparent and responsive; solicit and act on feedback; and offer support beyond the check.^27^ It shifts the focus from funding for “quick wins” and “low-hanging fruit” to “investing in long term outcomes,” ensuring that benefits reach those affected, and supporting efforts to tackle social and structural determinants of health as root causes of gendered health disparities.

**Outcomes**

To date, over 180 grant making organisations have signed the Trust-Based Philanthropy Project’s philanthropic commitment to trust based action,^28^ signalling a promising shift in the funding landscape. Early evaluations of Mackenzie Scott’s trust based funding approach, which has disbursed over $19bn to more than 2000 organisations and largely aligns with decolonial feminist recommendations, have shown that well supported non-profit organisations are now better placed to serve their communities.^29^ For those working towards gender equality and health equity, this represents a significant contribution towards improving health outcomes for those most in need of support and services.

Applying our three guiding questions—who is recognised as an innovator, what counts as an innovation, and who are we innovating for?—to the three aspects of innovation outlined above illustrate how more inclusive and equity centred approaches to innovation, aligned with decolonial feminist values, can lead to improved health outcomes by tackling social and structural determinants of health through innovations and interventions that are contextually informed and community driven.

## Innovation at a crossroads

Globally, health innovations have improved and saved billions of lives. Equally, stark disparities in health outcomes persist,^30^ exacerbated by intersecting markers of marginalisation and discrimination. Innovations that fail to centre local context and community priorities risk producing generic, “one-size-fits-all” solutions that fail to meet diverse needs, reinforce top-down decision making, and ultimately hinder progress towards genuine health equity. While definitions of social innovation that centre societal benefit are aligned with a decolonial feminist lens, critical questions remain about what innovations are introduced to which contexts—and more importantly, to what end.

The examples presented here challenge conventional power dynamics by broadening what we count as innovation and who we consider innovators. They facilitate more inclusive and just social innovation processes that tackle not only the symptoms of social and structural determinants of (ill) health, but their systemic and root causes as well.

What does the application of a decolonial feminist approach to innovation processes and products look like in practice? While each case study highlights new ways of working across a range of stakeholders, including governments, researchers, activists, community leaders, and funders, here we limit ourselves to three actionable recommendations as a starting point (table 1).

**Table 1:** Recommendations for implementing a decolonial feminist approach to innovation

Stakeholder group	Recommendation
Researchers	Adopt and implement a reflexive approach that fosters equitable partnership. Challenging internalised biases and assumptions is a key component of any process that seeks to ensures community based research and innovation considers the needs of communities. A reflexive process explores individual positionality and motivation, requiring an interrogation of power relations that embraces discomfort. A reflexive practice embraces continuous dialogue with others to facilitate collective learning and is intentional in harnessing insights to inform action, ensuring reflexivity leads to tangible change in methods, models, and modes of partnerships^31^
Policy makers	Look beyond the “usual suspects” to co-create policies that advance health for all. Policy and decision makers must take steps to create and enforce policies that meaningfully integrate and collaborate with knowledge holders beyond the usual suspects to support the creation of inclusive and just innovation environments. A decolonial feminist interrogation of who we consider experts and what we consider evidence, what biases are embedded within these notions, and how we can take an intentionally expansive approach will better serve the health needs and outcomes of all
Funders	Empower community led innovation through flexible, long term support. More flexible and longer term funding and investment models will benefit the development of women’s health innovations by fostering environments supporting experimentation, providing time and space to take risks, navigate failure, and iterate solutions. Immediate changes include longer grants that enable transformative solutions, core funding that supports change makers on the ground, reverse calls for proposals that prioritise community needs over funder interests,^32^ and multidirectional accountability mechanisms that meaningfully transform power dynamics

A decolonial feminist approach that questions who we recognise as experts, what we consider innovation, and whose interests we prioritise is key to driving significant, systemic, and structural changes towards delivering on the promise of health for all. At the individual and institutional level, acts of change such as the ones outlined above are an important first step. However, for the seismic shifts required in global health, we must work together at an ecosystem level to fundamentally reassess what the current system looks like and reimagine what it can and must look like.

Such change will not come uncontested: power protects itself, and any significant attempt to challenge vested interests often meets with resistance and retaliation. Sustained efforts are required to achieve our desired changes, but limited time, resources, and energy can make it challenging to focus on glacial shifts instead of quick wins. Collective action is necessary to overcome these barriers by leveraging shared strength and resources and, when guided by decolonial feminist values, ensure that we do not reproduce the very power asymmetries and structures we wish to dismantle.

**Key messages**

- Coloniality and patriarchy shape priorities, resources, decisions, and measures of success in health
- Overlooking these power systems sustains inequities and can make innovations ineffective or harmful
- A decolonial feminist lens can guide more equitable health innovation

## Notes

Contributors and sources TNA specialises in iterative decolonial feminist approaches for gender equality and health equity. AG is a Truwulway woman who leads a programme of research focused on respect and protection of indigenous cultural medicines knowledge. SOO has written and spoken widely on the need to rethink and reform global health and decolonise philanthropy. SA examines social inequities in health across the life course, with a focus on the wellbeing of refugees and labour migrants in the Arab region. ER co-led a regional social innovation consultancy and interrogates notions of expertise and evidence in health policymaking through a decolonial feminist lens. All authors co-conceptualised and contributed to this article and approved the final manuscript; TNA and ER co-wrote the first and final drafts. ER is the guarantor.

This article is part of a collection developed in partnership with the Gates Foundation. Open access fees were funded by the Gates Foundation. *The BMJ* commissioned, peer reviewed, edited, and made the decision to publish. Smruti Patel and Jocalyn Clark were the lead editors for *The BMJ*.
