# Decolonising women’s health innovation

**DOI:** 10.1136/bmj.s462

**Published:** 2026-03-10

Alana Gall’s affiliation with the Traditional, Complementary and Integrative Healthcare Coalition was omitted from the author details in this article by Tiffany Nassiri-Ansari and colleagues (*BMJ* 2025;391:e085683; doi:10.1136/bmj-2025-085683; 10 Oct 2025). The online version has been corrected.

